# Possible Biomarkers for the Early Detection of HIV-associated Heart Diseases: A Proteomics and Bioinformatics Prediction

**DOI:** 10.1016/j.csbj.2015.02.001

**Published:** 2015-02-18

**Authors:** Suraiya Rasheed, Rahim Hashim, Jasper S. Yan

**Affiliations:** Laboratory of Viral Oncology and Proteomics Research, Keck School of Medicine, University of Southern California, Cancer Research Laboratory Building, 1303 North Mission Rd, Los Angeles, CA 90033, USA

**Keywords:** HIV-cardiomyopathy, Ryanodine-receptors, ITPR, PI3K, Fetal-cardiac myosin, Myosin light-chain kinase

## Abstract

The frequency of cardiovascular disorders is increasing in HIV-infected individuals despite a significant reduction in the viral load by antiretroviral therapies (ART). Since the CD4 + T-cells are responsible for the viral load as well as immunological responses, we hypothesized that chronic HIV-infection of T-cells produces novel proteins/enzymes that cause cardiac dysfunctions. To identify specific factors that might cause cardiac disorders without the influence of numerous cofactors produced by other pathogenic microorganisms that co-inhabit most HIV-infected individuals, we analyzed genome-wide proteomes of a CD4 + T-cell line at different stages of HIV replication and cell growth over > 6 months. Subtractive analyses of several hundred differentially regulated proteins from HIV-infected and uninfected counterpart cells and comparisons with proteins expressed from the same cells after treating with the antiviral drug Zidovudine/AZT and inhibiting virus replication, identified a well-coordinated network of 12 soluble/diffusible proteins in HIV-infected cells. Functional categorization, bioinformatics and statistical analyses of each protein predicted that the expression of cardiac-specific Ca2 + kinase together with multiple Ca2 + release channels causes a sustained overload of Ca2 + in the heart which induces fetal/cardiac myosin heavy chains (MYH6 and MYH7) and a myosin light-chain kinase. Each of these proteins has been shown to cause cardiac stress, arrhythmia, hypertrophic signaling, cardiomyopathy and heart failure (*p* = 8 × 10^− 11^). Translational studies using the newly discovered proteins produced by HIV infection alone would provide additional biomarkers that could be added to the conventional markers for an early diagnosis and/or development of specific therapeutic interventions for heart diseases in HIV-infected individuals.

## 1 Introduction

The incidence of the acquired immunodeficiency syndrome (AIDS) has been successfully halted by a combination of antiviral therapies (ART), which suppress the human immunodeficiency virus (HIV)-load in the plasma of HIV-infected patients. However, despite the reduction of virus load, a significant number of HIV-infected individuals develop multiple immune-response and metabolic diseases including multiple cardiac disorders [1,3,21,26,31,32,38,45,49]. Global studies and Data Collection on Adverse Events of Anti-HIV Drugs (D: A:D) have indicated that although the traditional cardiovascular risk factors such as elevated lipid levels, diabetes and hypertension are common among both the HIV-infected and uninfected controls, a 26% increased risk of myocardial infarction and dyslipidemia could be associated to ART exposure [14,15,67]. This phenomenon was increased among women even when adjusted for other risk factors [17,62].

While many different cell types in the body are susceptible to HIV-replication, the bulk of the virus load in the blood comes from the peripheral mononuclear cells including CD4 + T-cells, macrophage and dendritic cells. Earlier studies by our group had demonstrated that infants who died of “sudden death” syndrome had significant quantities of HIV-infected T-cells in the heart, and HIV could be cultured from pericardial effusions of these infants [27]. Our earlier studies had also indicated that HIV-infected cells produce novel proteins/enzymes, which protect the HIV-infected cells from apoptotic pathways, enhance fatty acid synthesis and induce a wide range of metabolic abnormalities which are involved in increased dyslipidemia in HIV-infected individuals [56].

Adult postmortem studies have also indicated that HIV-infected-activated CD4 + T-cells are recruited in the left ventricular myocardium in hearts from HIV-infected subjects [36]. The HIV-infected, T-cells remain in an activated state due to constant antigenic stimulation in both treated and untreated individuals [2]. A subset of T-cells present in the heart could be stained by immunocytochemistry for cyclooxygenase 2 (COX-2) and inducible nitric oxide synthase, indicating that HIV-infected T-cells present in the heart played an important role in the progression of myocarditis [36].

The T-cell activation has been associated with dysregulation of intracellular Ca2 + signaling and cell survival with suppression of apoptotic pathways [4,8,9,33]. While only a brief spike of Ca2 + is needed for most normal cellular functions, an increased intracellular calcium (Ca2 +) is essential for the contraction and relaxation of heart muscles [6,28,64]. We therefore tested a hypothesis that HIV-infected/activated CD4 + T-cells express abnormal protein factors/enzymes that can infect heart tissue locally, and cause myocarditis, inflammatory responses and cardiac dysfunctions resulting in arrhythmia, cardiomyopathy and associated heart diseases.

In this report, we present experimental evidence that HIV-infection alone can enhance a well-coordinated expression of different types of Ca2 + release channels and kinases which have been shown to trigger overexpression of both fast and slow cardiac/fetal myosins together with a myosin light-chain kinase (MYH6-alpha, MYH7-beta and MYLK respectively) that are critical for the contraction and relaxation of heart muscles. Each of these proteins has been predicted by multiple bioinformatics tools including the binomial probability distribution to define the statistical significance of proteins associated with the development of arrhythmia, heart failures and cardiomyopathy in *HIV*-*uninfected* population groups (*p* = 8 × 10^− 11^). Inclusion of these unconventional biomarkers with the conventional risk factors would help in translational studies leading to early detection of HIV-associated cardiac diseases and possibly novel therapeutic interventions for specific disorders.

## 2 Study Design, Materials and Methods

### 2.1 Cells and Virus

A major consideration in the design of the present study has been to choose an *in vitro* system in which molecular changes in the signaling proteins solely due to long-term HIV-infection (at least 6–7 months) could be evaluated sequentially at different time points. While numerous cell types including the freshly isolated peripheral blood mononuclear cells (PBMCs) or cardiomyocytes can be infected with HIV *in vitro*, these cells are not suitable for long-term infections as they die due to cytotoxicity or apoptosis within 7–10 days of infection. Further, the cytopathic indices of HIV-infections in primary cells vary from person to person due to genetic heterogeneity of human population groups and differences in the susceptibility of different human cell types to HIV replication (Rasheed, personal observations). We also could not conduct proteomics studies directly in HIV-infected individuals without first identifying protein profiles of cells infected by HIV alone, because most HIV-patients are co-infected with other pathogenic viruses and microorganisms that may produce factors similar to those induced by HIV. We therefore chose a genetically stable, single-cell-clone of a human T-cell line (RH9) [55] and infected it with a biologically cloned North American HIV-Clade B (HBX) *in vitro*. The differentially-regulated proteins expressed in HIV-infected and counterpart uninfected cells were evaluated at numerous time points from 1.5-, 3,6-, 12- and 24-h, followed by weekly, then 4–6 weeks samplings for 6–7 months post-infection. In addition, proteomes were also studied from a chronically-infected RH9 cell line that has been maintained in our lab for decades as it can sustain HIV-replication for prolonged periods *in vitro* without exhibiting major cytopathic effects or apoptosis [54,56]. If these infected cultures showed excessive apoptotic cells due to overproduction of virus particles or viral proteins (gp120, Tat), in a crowded culture we harvested an aliquot of these cells for testing and added 1–2 × 10^6^ per ml uninfected RH9 cells from the same cell-stock to maintain virus infected cultures. The uninfected cells were added to the infected cells approximately after 2–3 months of continuously maintaining these cells *in vitro*. An advantage of this regimen of analysis was that both newly induced cellular proteins and those produced by chronically infected cells were detected reproducibly multiple times. If a protein was not detected at least twice, it was not included in the analyses.

### 2.2 Protein Isolation and Proteomics Analyses

We have used a rapid lysis method that was developed in our laboratory several years ago [54,56]. A sequential protein extraction technique has been extremely helpful in studying the dynamics expression of proteins associated with the plasma membrane, extracellular matrix, cytoplasm and nucleus. The membrane and extracellular matrix proteins were isolated by lysing cells for 15 s in solution containing 8 M Urea, 2% (*w/v*) CHAPS, 2% mercaptoethanol, 2.5% protease inhibitor cocktail, and 150 units/200 μl endonuclease. Each lysate was gently sonicated for 2 s then clarified first at 14,000 rpm followed by centrifugation at high speed (100,000 ×*g*) for 90 min. Soluble proteins in the *supernatants* were used to fractionate by 2-dimensional gradient (6–18%) gel electrophoresis (2DE). Cell-pellets were solubilized again with 9 M urea, 2 M thiourea and 4% CHAPS with other reagents being the same as above. This process was repeated one more time. More than 200 gels were evaluated and differentially expressed proteins from HIV-infected and uninfected controls cells were compared by using PDQuest software (BioRad). The presence or absence of each protein was confirmed by the matrix-assisted laser desorption ionization time of flight mass spectrometry (MALDI-TOF-MS). Only those gel-spots that reproducibly confirmed proteins at least two times by MALDI-TOF-MS were included in bioinformatics and statistical analyses. We also preferred to use MALDI-MS for this analysis than the liquid chromatography (LC) MS because proteins of interest could be reproducibly confirmed by re-sampling protein spots *manually* from multiple gels and both the high molecular weight proteins and small peptide fragments could be identified by MALDI-MS accurately. Thus, the specificity and reproducibility of membrane and extracellular matrix protein identifications were favored in this study over the total number of proteins (sensitivity) detected within a proteome.

### 2.3 Inhibition of Virus Replication

To validate protein expression profiles before and after infection, we inhibited chronic virus replication (in RH9 cell line that was infected for several months), by treating cells with a potent anti-reverse transcriptase agent, Zidovudine/AZT for 48 h *in vitro*. Protein profiles from HIV-infected, uninfected and AZT-treated and untreated counterpart cells were compared with each other by the use of a software (PDQuest-BioRad) and differentially regulated proteins in AZT-treated/-untreated cells, with and without HIV-infection were identified by mass spectrometry.

### 2.4 Bioinformatics and Statistical Analyses

Presence or absence of differentially expressed (upregulated, downregulated and *de novo* expressed) proteins, functional categorization, bioinformatics and statistical analyses were conducted by the use of multiple tools (Ingenuity Pathway Analysis (IPA) Systems, Expasy/UniProt), and various analytical programs available through the National Center of Biotechnology Institute (NCBI), and other public databases. The expected or E-values for all proteins associated with the calcium-release channels and cardiac myosins are from the Global Functional Analysis Program embedded in the IPA. The total number of proteins that have been shown to be statistically significance was also calculated by using the right-tailed Fisher Exact Test and IPA systems analyses.

## 3 Results

Replication of HIV in CD4 + T-cell line (RH9) was highly efficient and proteomes could be analyzed sequentially in the absence of excessive cytotoxicity or cell death. These proteins represented changes due to numerous phases of virus replication and cell growth over > 6 months. The protein expression profiles from HIV-infected cells were compared with counterpart uninfected cells (with and without treatment with antiretroviral drug Zidovudine/AZT) by subtractive proteomics analysis and differentially expressed proteins were categorized as follows: 1) *de novo* induced proteins were produced only by HIV-infected cells; 2) upregulated proteins are those that were expressed in uninfected control cells in small amounts but were increased in quantity (overexpressed) by HIV-replication and 3) proteins that are downregulated or their expression was completely turned off after HIV-infection. All differentially regulated proteins between HIV-infected and uninfected cells were identified in multiple gels and proteins that were not confirmed at least twice by mass spectrometry were discarded from the analyses.

Functional categorization of several hundred proteins identified 12 proteins that have been shown to be involved in regulating calcium (Ca2 +) homeostasis and affect heart muscle functions (MYH6 and MYH7) (Table 1). The Ca2 + regulating proteins (*n* = 9) were either upregulated or induced *de novo* post-HIV infection. Each of these proteins could be divided into distinct classes of Ca^2 +^-release channels, Ca^2 +^ regulatory kinases, Ca^2 +^-binding and Ca^2 +^ cycling proteins (Table 1). In addition, we have identified embryonic cardiac myosin heavy chains (myosin-6 and myosin-7) and light chain myosin kinase (MYLK) (*n* = 3) that were coexpressed in HIV-infected cells only but were not detected in any of the uninfected cells or in cells that were treated with AZT. These newly identified proteins have been localized to the extracellular matrix, plasma membrane or endoplasmic reticulum although some of these proteins have been shown to shuttle back and forth from the cytoplasm to other organelles including the nucleus. Full names, abbreviations and accession numbers of all proteins are according to the Swiss-PROT/Uni-PROT global databases (Table 1).

Bioinformatics and statistical analyses of each of the 12 proteins indicated that the calcium signaling proteins are critical for regulating Ca2 + influx and efflux; and cardiac myosins and myosin kinase are involved in cardiac morphogenesis in the embryo and are essential for controlling the contraction and relaxation of the heart muscle.

In the following sections, we have described the properties and functionalities of each of these 12 proteins and discussed their associations with T-cell activation, Ca^2 +^ regulation, Ca^2 +^ signaling and development of heart diseases. Each of these proteins has been *independently* linked to arrhythmia, heart failure, cardiomyopathy or sudden cardiac death in *HIV*-*uninfected* individuals (*p* = 6.1 × 10^− 3^ – 1.8 × 10^− 7^).

### 3.1 Dysregulation of Calcium-release Channels and Regulatory Kinase

Calcium is an essential element for life and its expression is controlled by extremely intricate mechanisms of Ca2 + release channels and pumps embedded in the plasma membranes and endoplasmic reticuli of all cell types in the body. While only small amounts of Ca2 + are needed for the sustenance of most cell types, the muscle and nerve cells utilize higher amounts of Ca2 + for contraction and relaxation. Among the main Ca2 + regulatory proteins, 9 were dysregulated during HIV-replication, and the significance of each of these proteins has been defined in relation to the cardiac functions by multiple bioinformatics and statistical tools.

#### 3.1.1 The Ryanodine Receptors or the Type-1 Calcium-Release Channels

**Fig. 1:**
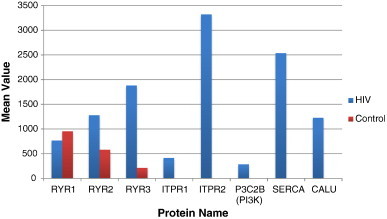
Proteomics analyses of Ca2 + release channels and signaling proteins; average quantity of each protein detected in HIV-infected cells (blue bars) versus uninfected counterpart controls (red bars). From left to right Type 1 Calcium Release Channels or Ryanodine Receptors 1,2 &3.: RYR1, 2 & 3 = ITPR1 and ITPR2 followed by Type 2 Calcium Release Channels, PI3K = phosphatidylinositol-4-phosphate 3-kinase C2 beta; SERCA = sarcoplasmic/endoplasmic-reticulum calcium ATPase2; CALU = Calumenin, a calcium-binding protein. ITPR1, ITPR2, PI3K, SERCA and CALU were expressed exclusively in HIV-infected cells and were not detected in any of the numerous counterpart uninfected cells tested. (For interpretation of the references to color in this figure legend, the reader is referred to the web version of this article.)

The type-1 calcium release channels include the ryanodine receptors RyR1, RyR2 and RyR3 that are responsible for the release of Ca 2 + intracellularly. While all three isomers of RyRs were detected in the HIV-infected and uninfected CD4 + T-cells, the expression of RyR1 was the same or very slightly downregulated (not significant) in HIV-infected cells compared to the uninfected control cells; and both RyR2 and RyR3 were upregulated post-HIV-infection (Fig. 1).

The RyR2 calcium release channel was detected more frequently (19 times) in HIV-infected T-cells compared to the uninfected counterpart cells in which it was detected only 5-times. RyR2 is the cardiac muscle-type receptor, which is essential for the regulation of the excitation–contraction coupling (ECC) or the contraction and relaxation rhythms of the heart muscle [5,11,12].The ECC depends directly on the opening and closing of RyR-Ca^2 +^ release channels at specific rates while these proteins also regulate systolic and diastolic functions of the striated cardiac and skeletal muscles [50,65,69]. RyR2 has also been shown to mediate arrhythmia and sudden-death syndrome [5]. RyR3 protein mobilizes stored Ca + 2 in both cardiac and skeletal muscle to initiate muscle contraction and dysregulation of both RyR2 and RyR3 that have been associated with cardiac diseases [11,12].

Whereas the expression of RyR2 and RyR3 has not been reported previously in relation to HIV-infection of T-cells, HIV infection of T-cells is activated due to increased intracellular Ca2 + levels [33]. An increased intracellular Ca2 + level in the muscles has also been shown to affect excitation–contraction coupling in cardiac muscle in experimental mice [16]. Our proteomics and bioinformatics analyses predict that the presence of HIV-infected-activated T-cells in the heart would enhance the release of Ca^2 +^ levels *in vivo* and a sustained overload of calcium would dysregulate expression of RyR2 and RyR3 in the heart. This would conceivably be predicted to induce oxidative stress and enhanced susceptibility to myocarditis, abnormal ECC, arrhythmia, ventricular dysplasia, heart failure and/or hypertrophy and cell death in HIV-infected individuals [20]. These functionalities are statistically significant (*p* = 5.0 × 10^− 3^).

#### 3.1.2 The Type-2 Ca^2 +^ Release Channels or the Inositol 1,4,5-Trisphosphate Receptors

The type-2 Ca^2 +^ release channels belong to a distinct class of inositol 1,4,5-trisphosphate receptors (ITPR1 and ITPR2; together known as IP3Rs). While these channels have been reported to be expressed on different types of human and animal cells including human T-cells [60,65], we have identified both ITPR1 and ITPR2 to be expressed exclusively in HIV-infected cells, and these receptors were not detected among several thousands of protein spots tested by mass spectrometry from the uninfected counterpart cells at different stages of cell growth overtime (Fig. 1). The ITPR1 enhances intracellular Ca^2 +^ stores and has been shown to interact with the HIV-encoded Nef protein in primary human peripheral mononuclear cells [37].

Inositol 1,4,5-trisphosphate (IP3) is a soluble factor that binds to IP3Rs present on the SERCA located in the endoplasmic reticulum (see Section 3.1.3 below). IP3 also triggers the release of Ca2 + into the cytosol and increases its quantity in the cell. Both the type 1 and type 2 classes of Ca^2 +^ release channels (*i.e.* RyRs and ITPRs) are located in close proximity of each other and therefore they are phosphorylated during T-cell activation in the channel pore and work in harmony to maintain and regulate cardiac rhythm [19,20,24,65]. Another study suggested that primary macrophages exposed to HIV-encoded Tat protein also release intracellular calcium from IP3-regulated calcium stores which increases production of TNF-alpha [40]. However, these studies have not reported detection of ITPR2, RyRs, Ca^2 +^ regulatory kinases, Ca^2 +^ binding proteins identified in the present research (Fig. 1). Thus, our bioinformatic analyses of the co-expressed type 1 and type 2 classes of Ca^2 +^ release channels (*i.e.* RyR2 and ITPR2) in the T-cells and overexpression of PI3K and Ca^2 +^-kinase (CamK11) (see below) and other Ca2 + signaling or Ca2 + binding proteins would be predicted to dysregulate Ca^2 +^ cycling in the heart of HIV-infected individuals (*p* = 10^− 4^).

#### 3.1.3 *Sarcoplasmic*/*Endoplasmic Reticulum Ca2*+ *ATPase2* (SERCA)

Sarcoplasmic/Endoplasmic Reticulum (SER) Ca2 + ATPase2 (SERCA2 or ATP2A2) was expressed exclusively in our experimentally HIV-infected T-cells and was not detected in any of the thousands of protein-spots tested from uninfected counterpart T-cells at various stages of cell growth (Fig. 1). SERCA are Ca2 + ATPase pumps involved in regulating Ca2 + release for cellular homeostasis in health and disease [13,22,39,63]. This highly specialized ATPase controls the amount of free calcium in cardiac muscle fibers via Ca^2 +^ release-channels that are located in SERCA [28,33,41].

The stored Ca^2 +^ is released from SERCA through two different classes of calcium-release channels described above (*i.e.* IP3R and RyRs). Under normal conditions, Ca^2 +^ is pumped into SERCA and is released by IP3R or RyRs. The ratio between the expression levels of ITPRs to RyRs is therefore important for cardiac functions. Depolarization of sarcoplasmic/endoplasmic reticulum membrane triggers the contraction of muscles and when the Ca^2 +^ pumps re-accumulate Ca^2 +^ in the store the muscles relax [28,41,50]. Thus, dysregulation of SERCA causes defective interactions with Ca^2 +^ release channels (RyRs or ITPRs), which results in a wide range of abnormalities in several organs including dysregulated contraction/relaxation cycles, dilated cardiomyopathy, heart failure and cardiac death [22] (*p* = 0.00615).

#### 3.1.4 Calcium/Calmodulin-dependent Serine–Threonine Protein Kinase Type II Alpha Chain

The calcium/calmodulin-dependent serine–threonine protein kinase type II alpha chain (CaMKII) was detected only once in HIV-infected cells but in none of the counterpart uninfected cells. While we have not included CaMK11 in any statistical analysis, our bioinformatics analyses suggest that the expression of CaMKII may have been *suppressed* in HIV-infected cells by the overexpression of protein kinase C (PKC) a highly efficient serine/threonine kinase that we described earlier to be expressed exclusively in HIV-infected cells [54]. PKC could also compensate the functions of CaMKII especially in the presence of PI3K and multiple other kinases that were expressed concomitantly in HIV-infected cells [54,56]. However, it has been shown that even extremely small quantities of CaMKII can activate RyR2 by phosphorylation, which has been implicated in arterial fibrillation, cardiac arrhythmia, cardiac hypertrophy and heart failure [35].

#### 3.1.5 Calumenin (CALU), a Calcium-Binding Protein

Calcium-binding proteins are critical for modulating Ca^2 +^ release through RyRs or IP3Rs [25]. Calumenin (CALU) was upregulated exclusively in HIV-infected T-cells and it was not detected in the uninfected cells (Table 1; Fig. 1). An important characteristic of CALU is that it contains six Ca^2 +^-binding motifs in the amino-terminus and a tetrapeptide His-Asp-Glu-Phe, both of which are essential for maintaining protein–protein interactions in SERCA [58,68]. CALU is normally expressed in the endoplasmic reticulum of many cell types as it is involved in protein-folding and sorting in SERCA [25]. Persistent contact of cardiac cells with Ca^2 +^-regulatory proteins such as CALU is therefore critical for modifying contraction-relaxation signals of the heart muscle (*p* = 0.0109) [58].

#### 3.1.6 Phosphatidylinositol-4-Phosphate 3-Kinase C2 Beta (P3C2B/PI3Kinase)

The phosphatidylinositol-4-phosphate 3-kinase C2 beta (P3C2B) was upregulated in HIV-infected T-cells (Fig. 1). This enzyme contains three critical protein–protein interaction domains (phosphoinositide-3 kinases (PI3Ks), C2 and phox-homology domain) that are important for regulating cell signaling, myocardial contractility and cell survival [47,48,52,70]. PI3K is an important enzyme for normal heart functions as it binds to its receptors ITPR1 and ITPR2, both of which were expressed only in HIV-infected but not in uninfected T-cells (Fig. 1). The overexpression of several different tyrosine kinases in HIV-infected cells raises intracellular calcium concentration by activating phospholipases which generate inositol 1,4,5-trisphosphate (ITP) [24], that binds to its receptors. Concomitant expression of PI3K and its interacting receptors (ITPR1 and ITPR2) in the HIV-infected T-cells are therefore predicted to alter myocardial functions through dysregulated Ca^2 +^ leaks in the heart [66].

### 3.2 Dysregulation of Cardiac Muscle Myosin Heavy Chains and a Light Chain Kinase

#### 3.2.1 Cardiac Muscle Myosin Heavy Chains Alpha MYH6 and Beta MYH7

**Fig. 2:**
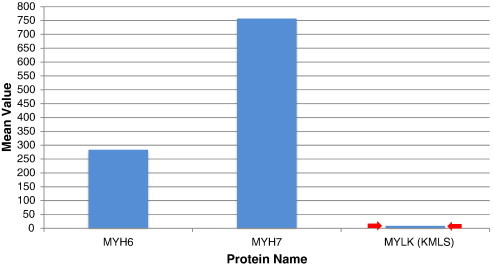
Mean values of cardiac myosins. Cardiac/fetal myosin 6 (heavy chain—fast ATPase), myosin 7 (heavy chain—slow ATPase) and myosin light chain kinase (MYLK). These cardiac myosins and associated kinase MYLK were expressed exclusively in HIV-infected cells (*i.e.* not detected in numerous samples tested from uninfected or AZT-treated infected and uninfected cells).

Myosins are molecular motors that form complexes with actin and move along the actin filaments while activating myosin ATPases and hydrolyzing ATP. These interactions convert chemical energy into mechanical forces essential for the contraction and relaxation of muscles [34]. While multiple myosins were expressed exclusively in HIV-infected T-cells, two of the most prominent cardiac related myosin proteins produced post-HIV-infection were fetal cardiac muscle myosin heavy chains alpha MYH6 and beta MYH7 (also known as MHC-alpha and MHC-beta respectively) (Fig. 2). These myosins were not detected in numerous samples examined from uninfected T-cells or in any of the HIV-infected cells treated with AZT. MYH6 is a “Fast” ATPase which hydrolyzes ATP in the heart and causes high-velocity muscle contraction. MYH7 is a “Slow” ATPase that is associated with slow-velocity muscle contraction. MYH6 also uses greater amounts of energy for shorter muscle contractions while MYH7 uses smaller amounts of energy for long-term contractions [34]. Thus, the cardiac energy levels depend on the amount of MYH6 and MYH7 expressed and a controlled regulation of these myosins is essential for maintaining a balanced level of the excitation–contraction coupling.

While the exact mechanisms by which these fetal genes affect cardiac functions are not well-understood, comparative studies in the coexpression networks of cardiac genes in the fetal (developing) myocardium, failing myocardium and cardiac hypertrophy, identified MYH6, MYH7 and SERCA to be the most critical fetal genes to be expressed in fetal heart and diseased tissues [10]. The fetal genes MYH6 and MYH7, are essential for the growth and morphogenesis of cardiac tissue during embryonic development; and an overexpression of both MYH6 and MYH7 has been shown to be significantly associated with atrial septal-defects, arrhythmia, cardiomyopathy, myocarditis, microvascular-dysfunctions, hypertrophy, and heart failure [7,18,46,51]. Both cardiac hypertrophy and heart failure have been associated with the re-activation of the set of fetal cardiac genes, which are suppressed postnatally and are substituted by the adult cardiac genes [10]. The levels of MYH6 and MYH7 expression have also been considered as markers for the severity of myopathy [23,30,43,53].

These observations and our bioinformatics analyses of differentially regulated proteins not only validate our hypothesis that HIV induces unique (fetal) genes MYH6 and MYH7 in chronically infected T-cells, but also demonstrate that the upregulation of both MYH6 (alpha MHC) and MYH7 (beta MHC) in HIV-infected cells causes an overabundance of Ca2 + and ATPase in the heart muscles, accounting for the cardiac stress and heart failure, particularly in patients whose hearts may be infiltrated with HIV-infected T-cells. These proteins could be predicted to be used as unconventional biomarkers of arrhythmia and cardiomyopathy.

#### 3.2.2 Myosin Light Chain Kinase

Myosin light chain kinase (MYLK) is a calcium/calmodulin-dependent enzyme which was co-expressed with MYH6 and MYH7 in association with multiple Ca2 + release channels described above. These proteins were expressed exclusively in HIV-infected cells but not in the numerous uninfected cells tested (Fig. 2). The calcium/calmodulin-dependent enzyme is involved in smooth muscle contraction via phosphorylation of myosin light chains and is essential in gap junction formation and permeability (*p* = 0.000440). MYLK also activates multiple cellular processes including muscle contraction, cell morphology, cell movement and it affects coronary, endothelial and microvascular dysfunctions.

Overexpression of MYLK has also been directly associated with numerous abnormalities in the vasculature as it compromises the integrity of endothelium in the coronary microvasculature [61]. Most importantly, the expression of MYLK is critical for transferring energy to MYH6 such that it can perform both smooth muscle and non-muscle functionalities and maintains basal levels of contractions via phosphorylation of cardiac myosin light chains.

### 3.3 Inhibition of HIV-Replication Prevents Expression of Myosins and Ca^2 +^ Signaling Proteins

To define the specificity of HIV-modulated proteins and to validate the relationship between HIV-replication and induction of Ca^2 +^ signaling and cardiac myosins, we treated both HIV-infected and uninfected RH9 T-cells with Zidovudine/AZT, a potent inhibitor of reverse transcriptase and HIV-replication [42,44]. Comparisons of proteomes from AZT-treated, -untreated, HIV-infected and -uninfected RH9 T-cells, indicated that both HIV-infected and uninfected cells treated with AZT had distinct profiles not related to HIV-infected or uninfected control cells. With the exception of small quantities of PI3K, none of the HIV-modulated myosins, Ca^2 +^ signaling cellular proteins or HIV-p24 antigen was detected in AZT-treated, HIV-infected cells. This is not surprising because PI3K is essential for cell-survival and it protects the AZT-treated cells (at least for short periods) from undergoing apoptosis [59]. These data indicate that HIV-replication was responsible for the modulation of multiple Ca^2 +^ regulatory proteins and enzymes that have been previously shown to be associated with cardiac muscle contraction.

### 3.4 Protein–Protein Interaction Pathways

**Fig. 3:**
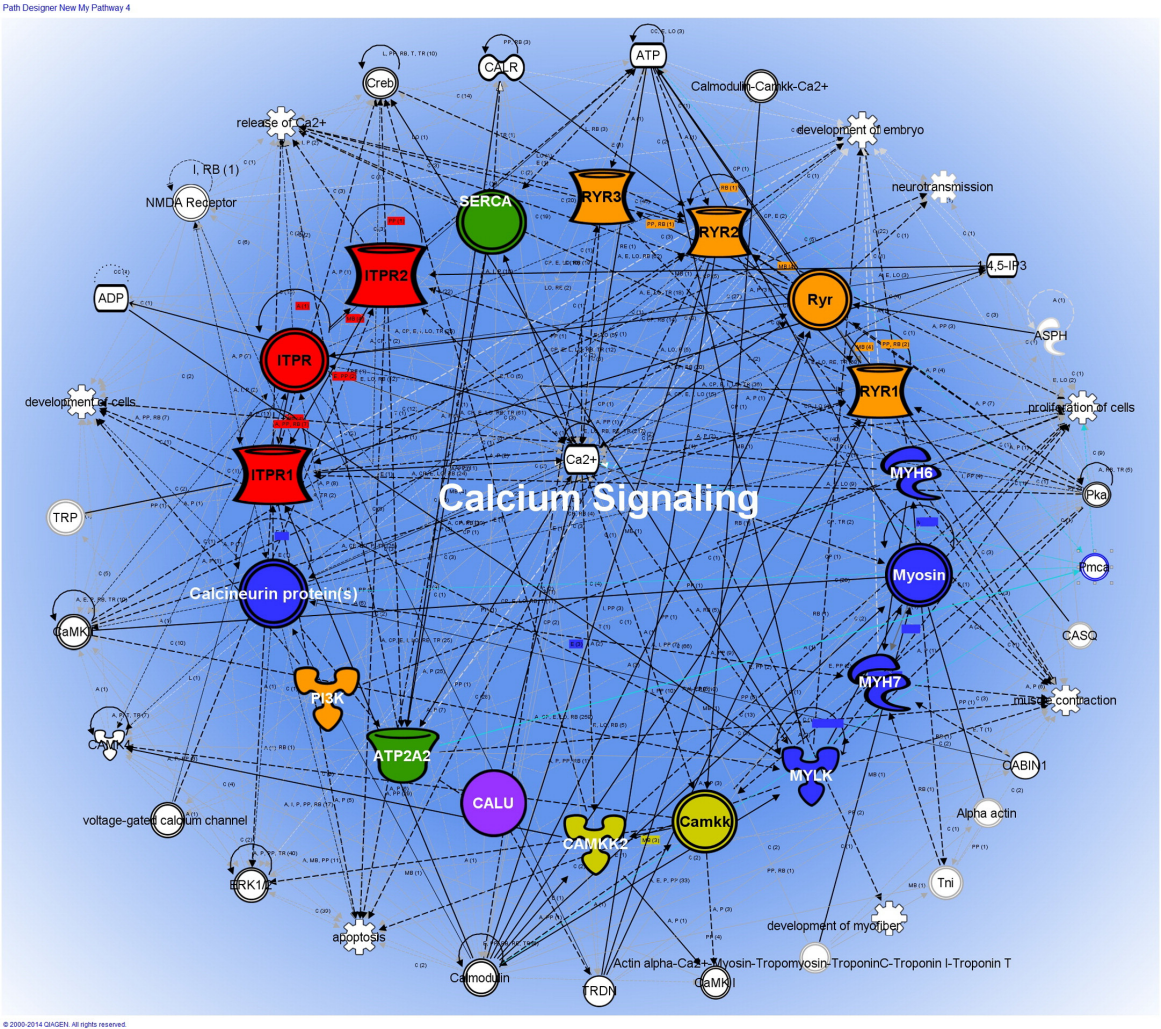
Calcium signaling and canonical pathways were constructed using Ingenuity Pathway Analyses System. Protein–protein interaction pathways showing a well-coordinated network of interactions between proteins that were dysregulated post-HIV-infection (RYRs) or expressed exclusively in HIV-infected cells. All 12 proteins identified to be expressed or overexpressed in HIV-infected cell were found within the calcium signaling protein–protein interaction networks. All proteins are highlighted in different colors and the symbols represent the different families described in the following diagram. (For interpretation of the references to color in this figure legend, the reader is referred to the web version of this article.)

To test if the newly discovered Ca^2 +^ related proteins are structurally and functionally related, we constructed protein–protein-interaction maps using Ingenuity Pathway Analysis (IPA) bioinformatics program. As seen in Fig. 3 all differentially regulated Ca^2 +^ receptors and Ca^2 +^ signaling proteins identified herein interact with each other and with numerous other cellular proteins along the Ca2 + canonical pathways that have been reported to be significantly associated with arrhythmia, cardiomyopathies and related disorders in different population groups (*p* = 2 × 10^− 16^).

The intracellular calcium-release and uptake are regulated by ion-channels that are located in the plasma membrane. Activation of membrane-bound receptors changes the membrane potential, which can alter protein–protein interaction pathways initiated at the plasma membrane to cytoplasm and the nucleus. We predict that abnormalities in Ca^2 +^ channels and dysregulation of Ca^2 +^-cycling proteins induce a sustained expression of cardiac fetal gene MYH6 and MYH7 which can then cause cardiac dysfunctions, hypertrophy and heart failure that have been linked to alterations in ECC, arrhythmia and cardiomyopathy.

## 4 Discussion

Most chronic diseases including cardiac disorders are multifactorial and numerous molecular pathways are operative in the development of these diseases. Our earlier studies had indicated that HIV replication enhances production of free fatty acids and many key proteins including low density lipoproteins that disrupt normal lipid metabolism [56]. In this report we have identified a well-coordinated network of cardiac receptors, RyR2 and RyR3, IP3Rs Ca^2 +^ release channels, Ca2 + signaling and regulatory proteins (Ca2 +-binding proteins, enzymes, and kinases) which induce fetal/cardiac MYH6 and MYH7 in chronically infected CD4 + T-cells. While the ITPR1 has been shown to interact with the HIV-encoded Nef protein in primary human peripheral mononuclear cells [37] and HIV-encoded Tat protein has been reported to increase IP3 and production of TNF-alpha in primary macrophages [40], none of the other proteins has been shown previously to be associated with HIV-infection.

Since HIV-infected CD4 + T-cells are present in the circulation and activated T-cells are routinely recruited to the heart, a perpetual stimulation of cardiac cells by abnormal Ca^2 +^ leaks by the expression of cardiac RyR2 and Ca^2 +^-regulatory enzymes, would be expected to weaken heart muscle progressively and is predicted to induce expression of cardiac fetal genes, MYH6 and MYH7. The co-expression of these fetal genes deteriorates cardiac muscle, and causes arrhythmia and cardiomyopathy (*p* = 2 × 10^− 16^).

Excessive Ca2 + release in the heart has also been shown to cause enhanced expression of numerous cytokines and chemokines post HIV-infection and these factors promote co-infections with other pathogenic viruses/microbes [29,57]. Further, numerous abnormal cellular and viral factors (HIV envelope-gp120 and Tat proteins) released in the hearts of HIV-infected individuals would cause oxidative stress locally and enhance susceptibility to myocarditis, arrhythmia, ventricular dysplasia and heart failure (*p* = 5.0 × 10^− 3^). Thus, most chronic diseases are related to multiple etiological factors that are produced by different cell types in the body which alter our immune responses and affect the entire body's metabolon.

While *in vitro* findings cannot be directly implicated to the development of diseases *in vivo*, there is no precedence of a coordinated expression of Ca^2 +^ release channels, Ca2 + regulatory enzymes and embryonic MYH6 and MYH7 genes/proteins in HIV-infected cells *in vitro or in vivo*. This could be because we studied the dynamics of protein expression over a long period of 6–7 months and identified proteins by a comprehensive and subtractive proteomics analyses in comparisons to the counterpart uninfected proteomes at different time points.

Our data have been supported by published reports in experimental animals in which MYH6 levels were significantly upregulated concurrently with the induced tachycardia and the left ventricular hypertrophy in mutant mice with familial hypertrophic cardiomyopathy [16]. While MYH6 and MYH7 have been associated with myocarditis, arrhythmias, heart failure, and related heart diseases in the non-HIV population groups (*p* = 8 × 10^− 11^), our studies have provide strong experimental evidence that HIV infection *alone*, without any co-infection or treatment can initiate an abnormal expression of novel myosin MYH6, MYH7 & MYLK proteins, involved in cardiomyopathy, arrhythmia and/or premature death (*p* = 0.000440).

Translational studies using the newly discovered Ca^2 +^ signaling proteins and cardiac/fetal myosins would lead to a better understanding of how a new combination of clinically significant biomarkers could potentially be used for early diagnosis of cardiac disorders and for the future development of targeted therapies. We propose that the specificity and sensitivity of early disease detection can be enhanced by profiling additional unconventional molecular markers including MYH6, MYH7, RyRs and ITPRs to study the progression of in both HIV-infected and uninfected individuals who are at a high risk for various cardiovascular events.

## Conflict of Interest

The authors declare that there is no conflict of interest with this project.

## Figures and Tables

**Table 1:** HIV-modulated proteins associated with cardiovascular functions and dysfunctions.

Protein name	Abbrev	Accession #	Function/diseases	*p*-Value
Myosin heavy chain, cardiac muscle alpha isoform	MYH6	P13533	“Fast” ATPase used to hydrolyze ATP in the heart and causes high-velocity muscle 2	0.000135
Myosin heavy chain, cardiac muscle beta isoform	MYH7	P12883	“Slow” ATPase used to hydrolyze ATP in the heart and causes slow-velocity muscle contraction	0.000440
Myosin light chain kinase, smooth muscle and non-muscle isozymes	MYLK	Q15746	Calcium/calmodulin-dependent enzyme involved in smooth muscle contraction via phosphorylation of myosin light chains; essential in gap junction formation and permeability	0.000440
Ryanodine receptor 1	RYR1	P21817	Malignant hyperthermia, central core disease (increased heart rate, respiratory insufficiency), arrhythmia, heart failure	0.000722
Ryanodine receptor 2	RYR2	Q92736	Heart failure, atrial fibrillation, catecholaminergic polymorphic ventricular tachycardia	0.000879
Ryanodine receptor 3	RYR3	Q15413	Abnormal contraction of skeletal muscle	0.00615
Inositol 1,4,5-trisphosphate receptor type 1	ITPR1 (or IP3R1)	Q14643	Myocardial hypertrophy, leaky channels, altered calcium signaling, contractile dysfunction and cardiac arrhythmias	0.00615
Inositol 1,4,5-trisphosphate receptor type 2	ITPR2 (or IP3R2)	Q14571	Cardiac hypertrophy, ventricular arrhythmia, atrial fibrillation	0.00615
Sarcoplasmic/endoplasmic reticulum calcium ATPase 2	AT2A2 (SERCA2)	P16615	Abnormal contraction/relaxation cycles, dilated cardiomyopathy, heart failure, cardiac death	0.00615
Calumenin	CALU	O43852	Ca2 + binding; regulates RYRs and coagulates blood	0.0109
Calcium/calmodulin-dependent protein kinase type II alpha chain	CaMKII	Q96RR4	Regulates SERCA-related Ca2 + release	Detected in small quantities
Phosphatidylinositol-4-phosphate 3-kinase C2 beta	PI3K (P3C2B)	O00750	Binds to its receptors ITPR1 and ITPR2; regulates myocardial contractility	0.00288

Table 1: Full names, abbreviations, accession numbers (UniProt) and functional significance of HIV-modulated proteins identified to be associated with cardiovascular functions dysfunctions. All 12 proteins are located in the plasma membrane/endoplasmic reticulum membrane. An overexpression of these proteins leads to cardiac muscle damage, tachycardia, heart failure, contractile dysfunction, cardiac hypertrophy, ventricular arrhythmia, heart failure and/or cardiac death.
